# αAsarone alleviates neuronal injury by facilitating autophagy via miR-499-5p/PDCD4/ATG5 signaling pathway in ischemia stroke

**DOI:** 10.3389/fphar.2025.1504683

**Published:** 2025-01-23

**Authors:** Yonghuan Yan, Linfang Wu, Lu Wang, Dandan Wang, Mengting Huang, Jinyong Peng, Yingying Huang

**Affiliations:** ^1^ School of Pharmacy, Anhui University of Chinese Medicine, Hefei, China; ^2^ College of Pharmacy, Dalian Medical University, Dalian, China; ^3^ Anhui Province Key Laboratory of Chinese Medicinal Formula, Anhui University of Chinese Medicine, Hefei, China; ^4^ Institute for the Evaluation of the Efficacy and Safety of Chinese Medicines, Anhui Academy of Chinese Medicine, Hefei, China

**Keywords:** autophagy, neuronal injury, αAsarone, stroke, miR-499-5p

## Abstract

**Introduction:**

αAsarone, an essential oil derived from *Acorus gramineus* Aiton, which has been successfully used to treat epilepsy in traditional chinese medicine, and has also been reported to confer neuroprotective effects on stroke. However, its mechanism of action remains poorly understood.

**Methods:**

The effects of αAsarone on autophagy were examined by WB, RT-qPCR, immunofluorescence colocalization, transmission electron microscope, and autophagic flux activity was measured by infecting HT22 cells with mRFP-GFP-LC3 adenovirus. And then, cells were transfected with both mimic-miR-499-5p and inhibit-miR-499-5p to investigate the role of miR-499-5p in regulating the effects of αAsarone on stroke. To further clarify the protective effect of αAsarone *in vivo*, TTC staining, neurological function score, H&E staining, Nissl staining, Laser speckle contrast imaging, transmission electron microscopy, immunofluorescence colocalization, WB and RT-qPCR were performed in the MCAO mice.

**Results:**

αAsarone was observed to inhibit the apoptosis of neuronal cells, and enhance autophagy. In addition, αAsarone promoted the expression of miR-499-5p. Targeting miR-499-5p can negatively regulate PDCD4 expression and the results from the dual-luciferase reporter assay demonstrate the direct targeting of PDCD4 by miR-499-5p. Promoting miR-499-5p can decrease the expression of PDCD4, increase ATG5, and enhance the protective effect of αAsarone on OGD/R injury while inhibiting miR-499-5p can weaken the effect of αAsarone. *In vivo* experiments further confirmed that αAsarone improved mice MCAO as evidenced by the amelioration of the neurological deficits and facilitated neuronal autophagy. Furthermore, we found that αAsarone reversed the effect of chloroquine, an autophagy inhibitor, and enhanced neuronal autophagy via miR-499-5p/PDCD4/ATG5 signaling pathway.

**Discussion:**

Our data suggest that αAsarone alleviates neuronal injury of stroke by facilitating neuronal autophagy through the miR-499-5p/PDCD4/ATG5 signaling pathway.

## Graphical Abstract

**Figure:**
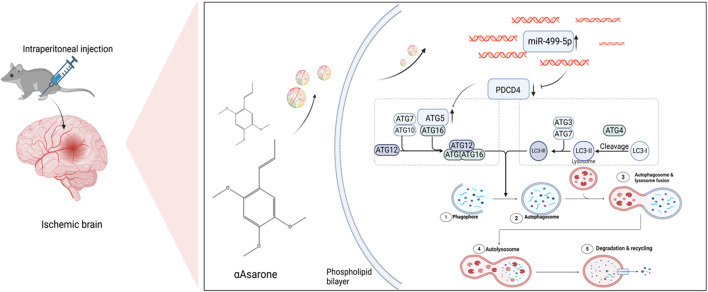
Schematic depicting the effect of αAsarone in stroke. Graphical Abstract was created using Biorender with permission for publication from Biorender.

## 1 Introduction

Stroke is, characterized by a high incidence rate, high disability rate, and high mortality. It is a catastrophic cerebrovascular event caused by burst/bleeding (hemorrhagic stroke) or cerebral vascular occlusion (ischemic stroke), leading to obstruction of blood flow to the brain, resulting in physical disabilities and various dysfunctions, which significantly threaten health and human life quality (Li et al., 2022; Shen et al., 2022). Ischemic stroke represents the majority of all types of strokes, comprising approximately 70%–80% (Luo et al., 2023). Thrombolytic therapy represents one of the most effective interventions for acute ischemic stroke, as it facilitates cell survival by restoring blood circulation to the ischemic region, however, there is a risk of bleeding following thrombolytic recanalization, which may further exacerbate brain damage (Zhang et al., 2022; Toljan et al., 2023). Notably, the complex pathological processes associated with stroke have been intensively studied in recent years, encompassing cellular acidosis, disturbances in energy metabolism, activation apoptotic genes, free radical production, intracellular calcium homeostasis, and autophagy (Wang et al., 2022; Beccari et al., 2023). Therefore, it is crucial to study the molecular mechanisms related to stroke progression and pathogenesis, as well as to find more effective drugs.

Autophagy is an evolutionarily conserved host cell self-destructive process characterized by the formation of double-membrane vesicles called autophagosomes that maintain cellular homeostasis and protect against pathogen invasion (Ammanathan et al., 2020; Jiang et al., 2021). Autophagy is important for maintaining cellular homeostasis within the brain, and recent research has demonstrated that it is also significant in various conditions, such as stroke (He et al., 2019; Kanno et al., 2022; Li et al., 2024). Furthermore, neuronal health is contingent upon the quality control functions of autophagy. Autophagy plays a crucial role as a metabolic process, autophagy is essential in clearing misfolded proteins under stress such as ischemia and hypoxia, senescent cells, and maintaining neuronal survival (Wang et al., 2019; Lin Z. et al., 2022; Zeng et al., 2022; Festa et al., 2023). Further understanding of how neuronal autophagy decreases neuronal death during stroke could unveil novel therapeutic directions for stroke management.

MicroRNAs (miRNAs) are small non-coding RNAs (19–25 nucleotides), play a role in regulating target messenger RNAs (mRNAs) after transcription. They achieve this control by binding to complementary sequences located in the 3′ untranslated region (3′-UTR) of the mRNA, which can lead to either mRNA degradation or inhibition of protein synthesis (Sun et al., 2020; Zhao et al., 2022). MiR-based therapeutics encompass a diverse array of mechanisms, including anti-oxidative stress, anti-inflammation, anti-apoptosis, pro-angiogenesis, blood-brain barrier protection, neuronal and axonal regeneration, anti-neurodegeneration, among other tissue remodeling (Lin L. et al., 2022; Qu et al., 2022; Yang et al., 2022; Wang et al., 2023). Research has shown that miR-499-5p has neuroprotective properties in brain injuries in rat pups, indicating its possible therapeutic use in managing such injuries (Jia et al., 2020). Based on our observations above, we hypothesized that miR-499-5p may play a role in stroke.

*Acorus gramineus* Aiton (plant names confirmed from http://www.theplantlist.org) is frequently utilized both independently and in conjunction with other herbs in traditional Chinese medicine. It is commonly used for the treatment of epilepsy and other neuropsychiatric diseases, and has been employed in the clinical practice of traditional Chinese medicine for thousands of years, such as Ditan decoction (Rajput et al., 2014; Acosta-Quiroga et al., 2021; Xu et al., 2022; Tao et al., 2023). Furthermore, *Acorus gramineus* Aiton has numerous health benefits, including neuroprotection, antihypertensive, anticonvulsant, antioxidant, antidepressant, anti-inflammatory, immunomodulatory activity, cardioprotective effects, and potential benefits for obesity (Rai et al., 2023). αAsarone, a primary component of volatile oil extracted from *Acorus gramineus* Aiton and that can pass through the blood-brain barrier, has demonstrated significant anti-stroke effects (Ge et al., 2022; Jiang and Hu, 2022). In our earlier studies, we found that αAsarone has a neuroprotective effect in a cell oxygen-glucose deprivation/reperfusion (OGD/R) model by mitigating oxidative stress and reducing cell apoptosis (Zhao et al., 2023), indicating that αAsarone has a positive impact on stroke. In the present study, we examined the role of αAsarone in alleviating stroke injury through the miR-499-5p signaling pathway.

## 2 Materials and methods

### 2.1 Regents

Nimodipine was purchased from Hunan Baicao Pharmaceutical Co., Ltd. (Hunan, China). Chloroquine (CQ) was purchased from MCE (Shanghai, China). αAsarone was purchased from Yuanye biological company (Shanghai, China), with purity ≥98%. SYBR Green Real-Time PCR Master Mix and gDNA remover ReverTra qPCR RT Master Mix were provided by Toyota CO., Ltd., located in Toyota, Japan. Meanwhile, the BCA Protein Assay Kit was sourced from Biosharp Labgic Technology CO., Ltd., based in Hefei, China. Antibodies for ATG5, LC3I, and LC3II were obtained from Proteintech. The PDCD4, LAMP1, goat anti-mouse and goat anti-rabbit were offered by Chengdu Zhengneng Biology Co., Ltd. (Chengdu, China).

### 2.2 OGD/R model establishment *in vitro* and drug treatment

The HT22 cell line was perserved in DMEM media enriched with 1% penicillin-streptomycin and 10% fetal bovine serum, incubated at 37°C in a 5% CO2 atmosphere, change cell culture medium daily. When the cells reached a confluence level of 60%–70%, they were subjected to hypoxic conditions by being rinsed with phosphate-buffered saline (PBS) and then underwent OGD for a duration of 6 h. Sugar-free medium was then replaced with complete culture medium and cells were reoxygenated for 24 h under standard oxygen levels. The HT22 cells were subsequently categorized into 6 different groups: Control, OGD/R group, OGD/R + Nimodipine group, and three groups with αAsarone (5, 10, and 20 nM). Therefore, 5, 10, and 20 nM were chosen as Low (L), Medium (M), High (H) concentrations in the flowing experiments. The mechanism part was divided into 8 groups: Control, OGD/R, OGD/R + αAsarone (20 nM), Inhibit-Control, Inhibit-miR-499-5p, Inhibit-miR-499-5p + αAsarone (20 nM), Mimic-Control and Mimic-miR-499-5p.

### 2.3 Cell viability measurement

HT22 cells were inoculated at a density of 1 × 10^7^cells/cm^2^ into 96-well plates. Following a 6-h interval of OGD, the cells received treatment with αAsarone after 12 h. The evaluation of cell viability using the non-radioactive CCK-8 assay (Biosharp, Jiangsu, China), adhering to the manufacturer’s guidelines, with absorbance levels recorded at 450 nm. Each experiment was executed in triplicate.

### 2.4 Autophagy flux assay

HT22 cells were seeded on 35 mm dishes featuring a glass bottom at a density of 1 × 10^7^ cells and subsequently transfected with mRFP-GFP-LC3 tandem fluorescent lentivirus. The next day, replace the medium with 2 mL of fresh medium. After 72 h post-transfection, the neurons were subjected to OGD/R for a duration of 6 h, with or without the addition of PNU282987 (100 μM). Additionally, Autophagosomes (GFP-positive, mRFP-positive) and autolysosomes (GFP-negative, mRFP-positive) within the neurons were visualized using confocal microscopy (Olympus, Tokyo, Japan). The Spots (>1 µm) per cell were conducted.

### 2.5 Dual luciferase reporter assay

Luciferase reporter vectors, namely miR-499-5p-wt and PDCD4-wt, were constructed by Hanheng in Hunan, China. Mutant vectors, miR-499-5p-mut and PDCD4-mut, were generated by modifying the gene sequences at the predicted binding sites. Cells were plated in 12-well plates at a suitable density. Following this, all plasmids were introduced into the cells and allowed to incubate for 24 h. Upon completion of the transfection period, the cells were collected, and the activity of firefly luciferase was assessed utilizing a dual-luciferase reporter assay system. Subsequently, the firefly luciferase activity was normalized against the activity of renilla luciferase.

### 2.6 Animals

Adult male specific pathogen-free (SPF) Sprague-Dawley mice, weighing between 20 and 24 g, were obtained from GemPharmatech Co., Ltd. The mice were housed under SPF-grade conditions for 1 week, during which they had unrestricted access to food and water. All experiments involving animals were performed following the guidelines set forth by the National Institutes of Health regarding the management and utilization of laboratory animals (NIH Publications No. 8023, revised 1978). The Animal Ethics Committee at Anhui University of Traditional Chinese Medicine (Hefei, Anhui, China) provided approval for these studies (ethics number: 2023149), which aimed to reduce pain, suffering, and distress. Before the experiments commenced, the mice experienced a fasting duration of 12 h.

### 2.7 MCAO model establishment and drug administration

The establishment of a stroke model in mices was conducted using an altered method of middle cerebral artery occlusion (MCAO), utilizing 3% isoflurane for anesthesia in adult male SPF mice. During the surgical procedure, the body temperature was maintained at 37°C. An incision along the midline of the neck was created to gain access to and isolate the common carotid artery, the external carotid artery, and the internal carotid artery. Nylon sutures were then placed approximately 10 mm deep from the common carotid artery to the internal carotid artery. After a duration of ischemia lasting 1 h, the nylon suture was taken out to promote reperfusion. In the sham group, the identical surgical procedure was carried out, but the nylon suture was not inserted. After 1 h of stroke, behavioral assessments were conducted using the Zealonga scoring method (Longa et al., 1989). The evaluation criteria are as follows. 0 points: There are no symptoms of neurological disorder and the patient’s functions are normal. 1: Unable to fully extend the contralateral forepaw. 2: Contralateral hemiplegia with crawling and turning. 3: The body leans toward the hemiplegic side when walking. 4: Unable to walk alone, unconscious; 5: death. The mice were subsequently randomized into seven groups: Sham, Model, αAsarone (10 and 40 mg/kg), Nimodipine (1.6 mg/kg), CQ (80 mg/kg), and CQ combined with αAsarone (40 mg/kg). Each drug was injected intraperitoneally into mice once for 3 consecutive days.

### 2.8 TTC staining

The assessment of the brain infarct volume was conducted with the use of 2,3,5-triphenyltetrazolium chloride (TTC) staining technique. Five coronal slices of the brain, each measuring 1 mm in thickness, were treated with 1.5% TTC solution. For analyzing the volume of the infarct, ImageJ software was utilized.

### 2.9 H&E staining and nissl staining

Following fixation, dehydration, and embedding in transparent paraffin wax, brain tissue specimens were sectioned to a thickness of approximately 4 µm. Hematoxylinand eosin (H&E) staining was then performed, allowing for the examination of pathological changes in the CA1 region of the hippocampus under a 400-fold magnification. The tissue sections were then immersed in Nissl solution at 50°C for a duration of 10 min, followed by immersion in 95% alcohol. After rinsing with distilled water, the slices were transferred to 70% alcohol. Finally, xylene was added before sealing the container.

### 2.10 Immunofluorescence

After fixation, embedding, preparation, permeabilization, and blocking, brain specimens underwent treatment with primary antibodies (MAP2 and LC3) at a temperature of 4°C overnight. Following three rinses in 0.02 M PBS, each lasting 10 min, the specimens the specimens were exposed to the secondary antibody at 37°C for a duration of 1 h. The samples were then rinsed again three times for 10 min with 0.02 M PBS, after which the nuclei were then labeled with DAPI for 2 h at 37°C, confocal laser scanning microscopy (Nikon, Tokyo, Japan) was utilized to scan the sections and dishes.

*In vitro*, HT22 cells underwent incubation with primary antibodies (LAMP1 and LC3), and the remaining steps were performed as previously outlined.

### 2.11 Transmission electron microscopy (TEM)

Ultrastructural changes in brain tissues and HT22 cells were evaluated using TEM. The hippocampi were fixed overnight at 4°C in a 2.5% glutaraldehyde solution. The ultra-thin sections were then subjected to staining with lead citrate and uranyl acetate before being observed and imaged with atransmission electron microscope (TEM; Tokyo, Japan).

### 2.12 Western blot analysis

Hippocampal cells were homogenized in cold RIPA buffer with PMDF for a duration of 10 min, both *in vivo* and *in vitro*, and total protein was extracted through high-speed centrifugation. After quantifying the protein using the BCA assay, 30 μg of the extract was loaded onto 10% or 12% (w/v) SDS-PAGE gels for 1.5 h of electrophoresis, after which the proteins were conveyed onto a polyvinylidene fluoride membrane for about 1.5 h. The membranes were incubated in a solution of 5% skim milk for 1.5 h at a temperature of 37°C. Afte three washes of 10 min each using Tris-buffered saline containing Tween 20. The membranes were then treated with primary antibodies LC3 (1:2,500), LAMP1 (1:1,000), PDCD4 (1:1,000), ATG5 (1:1,000), and GAPDH (1:1,000) for a period of 8 h at 4°C. After this incubation, the membranes were washed three more times before being exposed to either goat anti-rabbit IgG (1:10,000) or goat anti-mouse IgG (1:10,000) in Tris-buffered saline containing Tween (TBST) at room temperature for a duration of 2 h. Once more, three additional washes with TBST were carried out, each lasting 10 min, after which enhanced chemiluminescence solution and the ImagerJ software were employed to visualize protein expression on the membrane.

### 2.13 Real-time reverse transcription-quantitative PCR

Isolate RNA from ischemic brain homogenate or HT22 cells using RNA extraction reagents. Primers for miR-499-5p, PDCD4, ATG5, LC3I, LC3II, LAMP1, and β-Actin were designed by Sangon Biotech Co., Ltd. (Shanghai, China), and the sequences of these primers can be found in Table 1. The RT-qPCR process followed the protocols described in earlier studies (Ponsford et al., 2021).

**TABLE 1:** Primer sequence of PCR.

Gene	Sequence
β-Actin	Forward: 5′- AGGAAGGACCTGTATGCCAACA-3′ Reverse: 5′- GCGCGGTGATCTCTTTCTG-3′
LAMP1	Forward: 5′- CGTCCAGCTCATGAGTTTTGT-3′ Reverse: 5′- AGACTGGGGTCAGAAGTGTTC-3′
LC3I	Forward: 5′- GCATCCAAACAAAATCCCGGTC-3′ Reverse: 5′- AAGCCATCCTCATCCTTCTCCT-3′
LC3II	Forward: 5′- AGTGAAGTGTAGCAGGATGA-3′ Reverse: 5′- AAGCCTTGTGAACGAGAT-3′
PDCD4	Forward: 5′- AAGCCATCCTCATCCTTCTCCT-3′ Reverse: 5′- GTCACCCCTAAATGCCACCG-3′
ATG5	Forward: 5′- GTCAGATCCGCTAGAGATCTGCTTACTAAGTTTGGCTTTGGTT -3′ Reverse: 5′- GATATCTTATCTAGAAGCTTAAGGGTGACATGCTCTGATAAAT -3′
miR-499-5p	Forward: 5′- ACTGCTTAAGACTTGGAGTGA-3′ Reverse: 5′- TACATTGGTGTCGTGGAGTCGGCAA-3′
U6	Forward: 5′- ATTGGAACGATACAGAGAAGATT-3′ Reverse: 5′- GGAACGCTTCACGAATTTG-3′

### 2.14 Statistical analysis

Data are presented as the mean ± standard deviation (SD) derived from a minimum of three independent experiments. Statistical analysis was performed using one-way analysis of variance (ANOVA), followed by the least significant difference *post hoc* test for multiple comparisons, utilizing GraphPad Prism version 9.0 software. A significance level of P < 0.05 set to determine statistically significant differences.

## 3 Results

### 3.1 αAsarone protects HT22 cells from OGD/R injure

**FIGURE 1:**
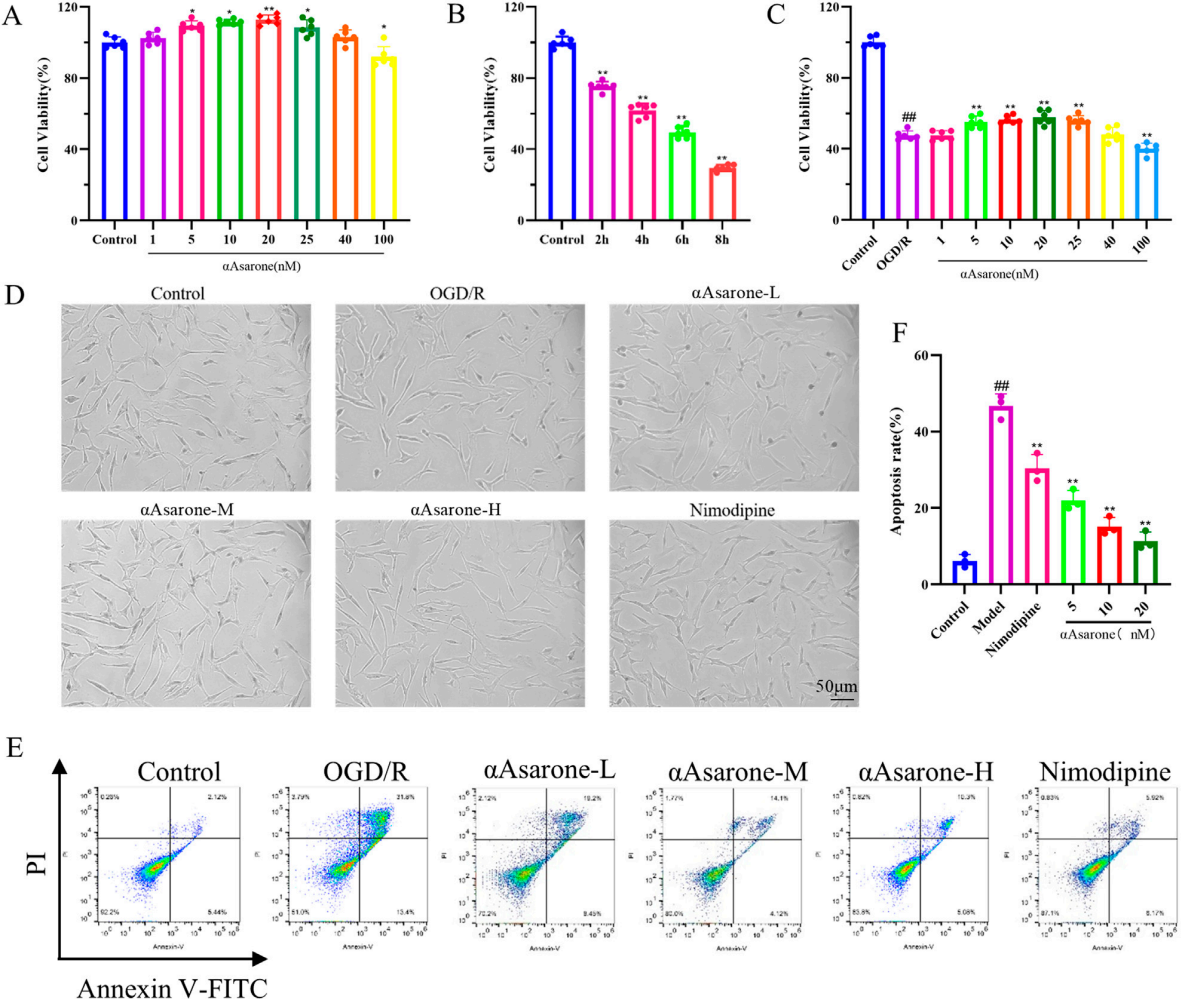
αAsarone protects HT22 cells from damage caused by OGD/R. **(A)** Safe concentration of αAsarone (n = 6). **(B)** Modeling concentration of OGD/R (n = 6). **(C)** Effective protective concentration of αAsarone (n = 6). **(D)** Cell morphology was observed by biomicroscopy. The concentrations of αAsarone-L, αAsarone-M, and αAsarone-H are 5 nM, 10 nM, and 20 nM, respectively. **(E, F)** Flow cytometry detection of cell apoptosis (n = 3). The data are presented as mean ± SD. Statistical analysis showed ^##^ *P* < 0.01 vs. the control group, *P < 0.05, **P < 0.01 vs. the OGD/R group.

To establish the safe and protective concentrations of αAsarone, HT22 cells received treatment across various concentrations: 1, 5, 10, 20, 25, 40, and 100 nM. We observed an increase in proliferative ability at 5, 10, and 20 nM (*p* < 0.01) (Figure 1A), Four time points (2 h, 4 h, 6 h, and 8 h after OGD/R) were selected for observation. We found that after 6 h of OGD/R, which was statistically significant when compared to the control group, the cell survival rate was approximately 50%, (*p* < 0.01) (Figure 1B), indicating substantial toxicity to cell growth. Therefore, 6 h of OGD/R was selected as the modeling condition. Subsequently, treatment of HT22 cells with αAsarone for 12 h revealed that 5, 10, and 20 nM enhanced cell survival rates compared to the model group (*p* < 0.05) (Figure 1C). The protective effects of αAsarone on OGD/R-induced HT22 cells were evident, with the most pronounced effects noted at dose-dependent increases at 5, 10, and 20 nM. Therefore, 5, 10, and 20 nM were chosen as Low (L), Medium (M), High (H) concentrations in the flowing experiments. Additionally, After the OGD/R cells were treated with αAsarone, their morphology improved and the number of adherent cells increased (Figure 1D). Additional examination of cellular apoptosis through flow cytometry revealed a significant increase in neuronal apoptosis post-OGD/R, which was effectively reversed by αAsarone at concentrations of 5, 10, and 20 nM (Figures 1E, F). These findings suggest that αAsarone alleviates cell injury induced by OGD/R.

### 3.2 αAsarone activated the autophagy in HT22 cells

**FIGURE 2:**
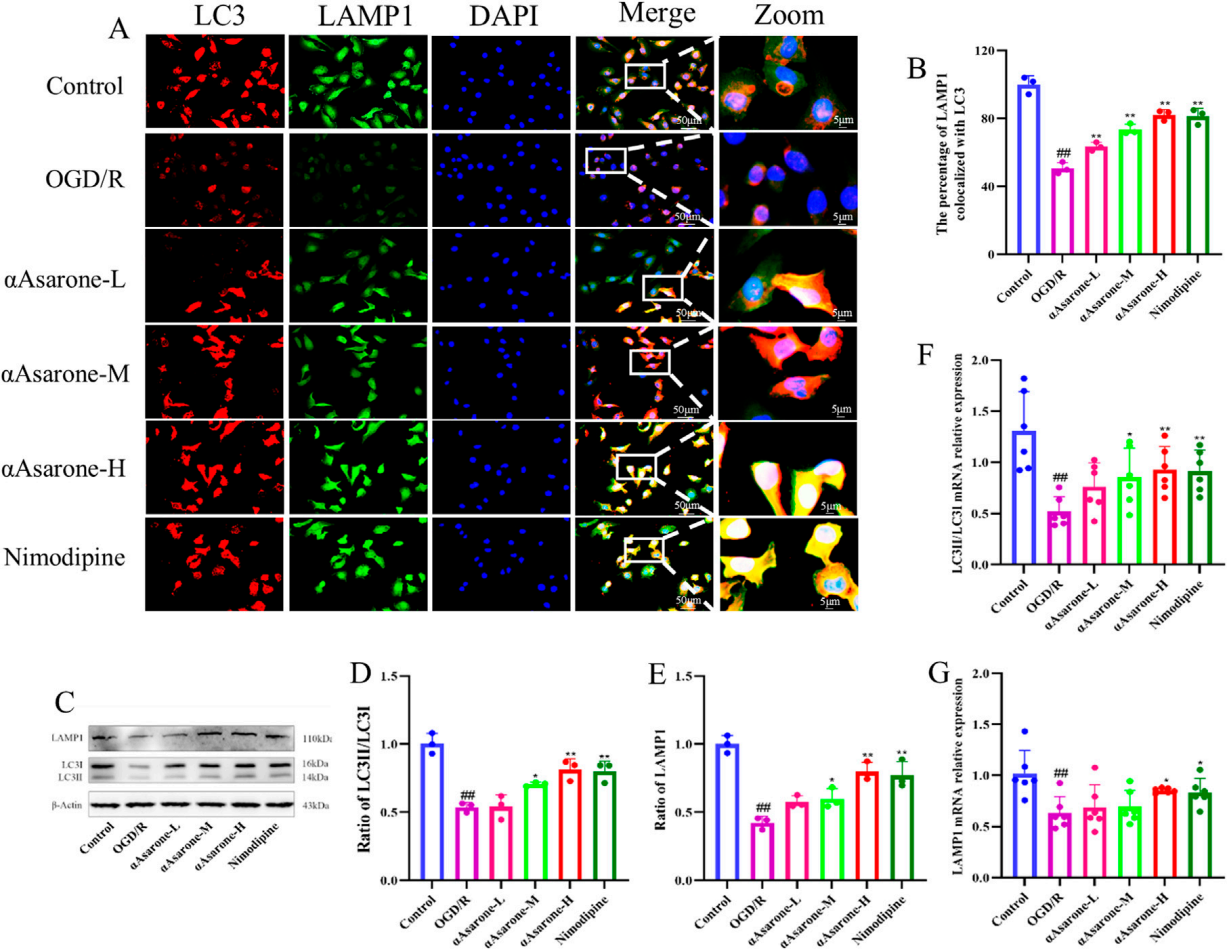
αAsarone activated the autophagy in HT22 cells. **(A)** Representative images of fluorescent staining for DAPI, LAMP1 and LC3 in the HT22 cells. Scale bar = 50 µm. Green puncta: LAMP1, red puncta: LC3, bule puncta: DAPI (n = 3). **(B)** The percentage of LAMP1 colocalized with LC3. **(C–G)** The western blots and RT-qPCR of LAMP1and LC3, as well as the quantitative analysis. All data are expressed as mean ± SD, Statistical analysis showed ^##^ *P* < 0.01 vs. the control group, *P < 0.05, **P < 0.01 vs. the OGD/R group.

To explore the effect of αAsarone on neuronal autophagy, we evaluated its effects on the expression levels of crucial proteins and genes, including LC3 and LAMP1, usin western blotting, RT-qPCR and fluorescent staining techniques. As shown in Figures 2A, B, double immunofluorescence staining was conducted for LAMP1, a marker of lysosomes, and LC3, an autophagy marker, to assess the potential effect of αAsarone on autophagy activation (measured by the percentage of LAMP1 that colocalizes with LC3) (Matarrese et al., 2014). The findings demonstrated that αAsarone markedly enhanced the colocalization of LAMP1 and LC3, indicating an increased rate of autophagy in the αAsarone-treated group. Furthermore, western blot and RT-qPCR analyses corroborated these findings, revealing elevated ratios of LC3-II/LC3-I and LAMP1 in the presence of αAsarone following OGD/R (Figures 2C–G), thereby suggesting that αAsarone activates autophagy.

### 3.3 miR-499-5p negatively targets and regulates PDCD4 in OGD/R cells

**FIGURE 3:**
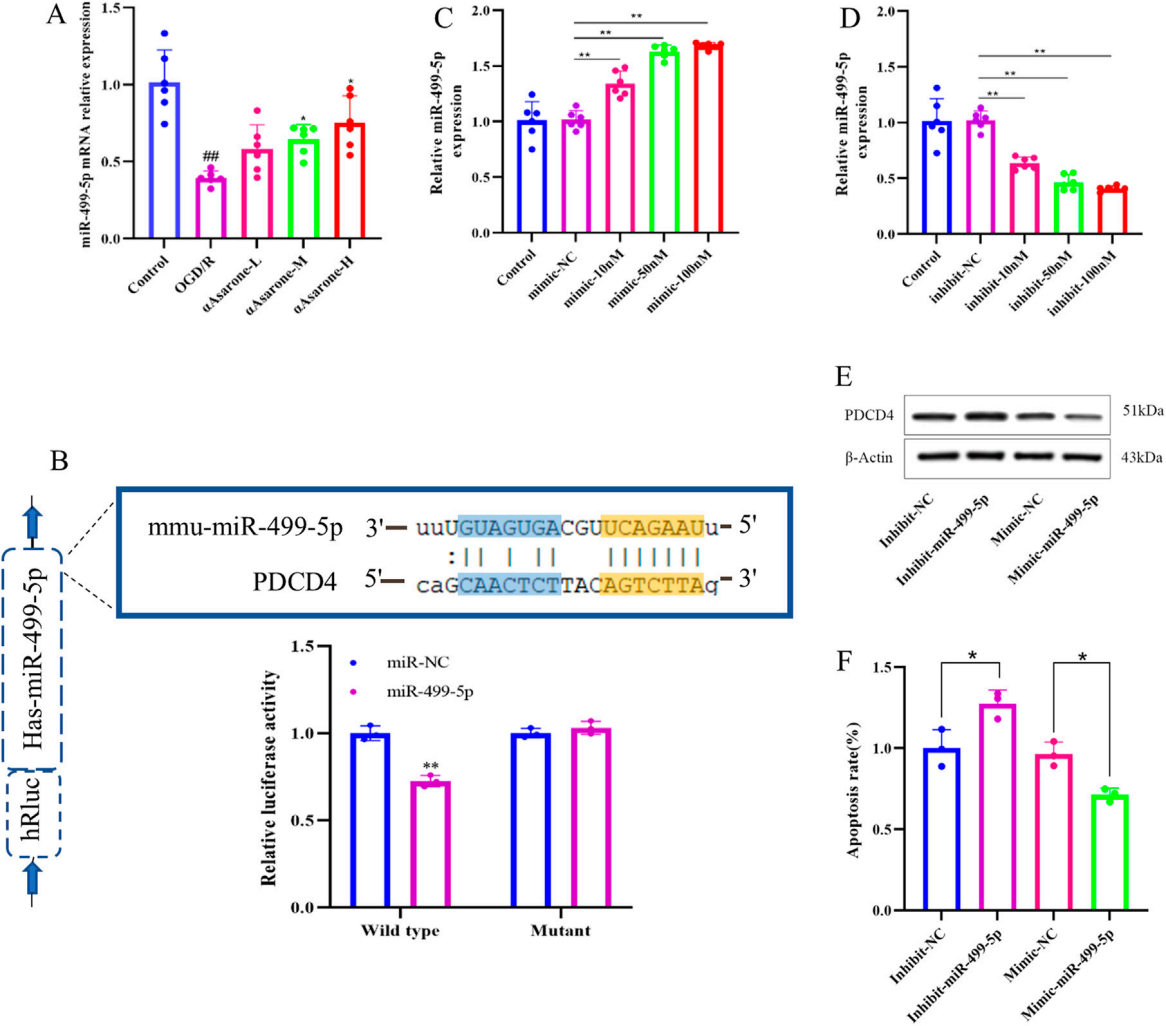
miR-499-5p in stroke. **(A)** miR-499-5p expression levels in HT22 cells after OGD/R injury (n = 6). **(B)** Dual luciferase reports. **(C, D)** Quantitative RT-qPCR experiment to confirm the efficacy of miR-499-5p mimics and inhibits transfection (n = 6). **(E, F)** Relative protein expression of PDCD4 from four groups containing miR-499-5p inhibitor, miR-499-5p mimics group and their corresponding control groups was evaluated through western blot (**P < 0.01 vs. the Inhibit NC group, *P < 0.05 vs. the Mimic NC group). Other data are expressed as mean ± SD, Statistical analysis showed ^##^ *P* < 0.01 vs. the control group, *P < 0.05, **P < 0.01 vs. the OGD/R group.

Following OGD/R injury, the expression levels of miR-499-5p in HT22 cells was observed to decrease after 6 h, Further, it was found that αAsarone enhanced the expression level of miR-499-5p in a dose-dependent manner. (Figure 3A, *P* < 0.01). Luciferase reporter gene assays demonstrated a reduction in luciferase activity in HT22 cells that were co-transfected with miR-499-5p mimics and the wild-type PDCD4 plasmid. Conversely, co-transfection with the mutant PDCD4 plasmid did not yield a significant effect, suggesting a direct physical interaction between PDCD4 and miR-499-5p (Figure 3B). Subsequent transfected with miR-499-5p inhibitors and mimics led to a significant alteration in miR-499-5p expression within HT22 cells and transfection efficiency reached 70% at a concentration of 50 nM, which was therefore selected as the concentration for subsequent transfections (Figures 3C, D). Western blot analysis indicated that PDCD4 was upregulated in HT22 cells transfected with the miR-499-5p inhibitor, whereas it was downregulated in cells transfected with the miR-499-5p mimic (Figures 3E, F).

### 3.4 miR-499-5p/PDCD4/ATG5 mediated the activation of autophagy by αAsarone

**FIGURE 4:**
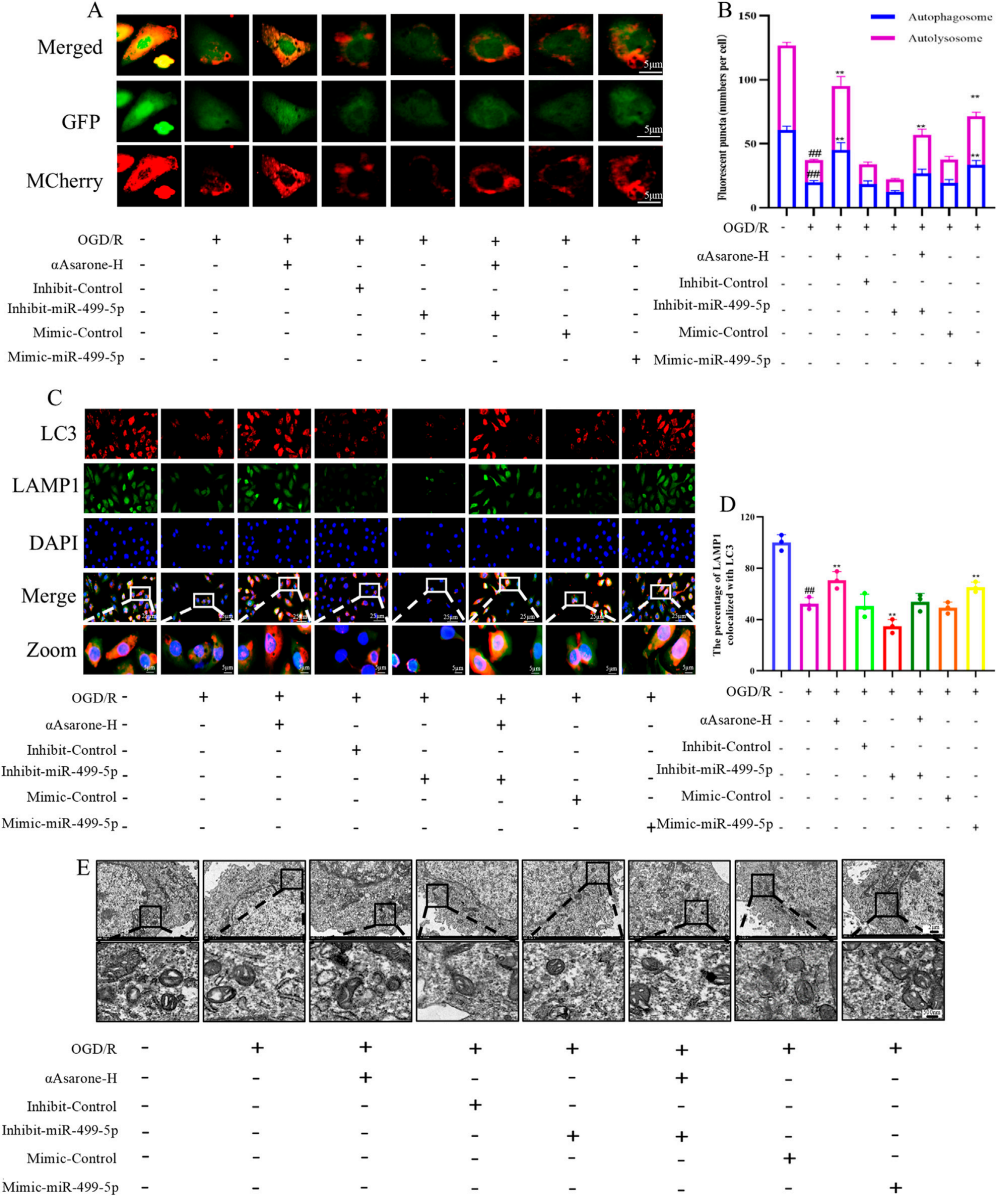
miR-499-5p/PDCD4/ATG5 mediated the activation of autophagy by αAsarone. **(A)** Autophagy flux assay indicating that miR-499-5p inhibitors blocked autophagy flux (n = 3) **(B)** Autophagy flow analysis revealed Autolysosomes represented by red dots and Autophagosomes represented by yellow dots in the Merge diagram. **(C)** Representative immunofluorescence staining for DAPI, LAMP1 and LC3 in the hippocampal neurons (n = 3). **(D)** The percentage of LAMP1 colocalized with LC3. **(E)** The representative electron micrographs in the HT22 cells were observed by TEM. Scale bar = 500 nm. All data are expressed as mean ± SD, Statistical analysis showed ^##^ *P* < 0.01 vs. the control group, **P < 0.01 vs. the OGD/R group.

**FIGURE 5:**
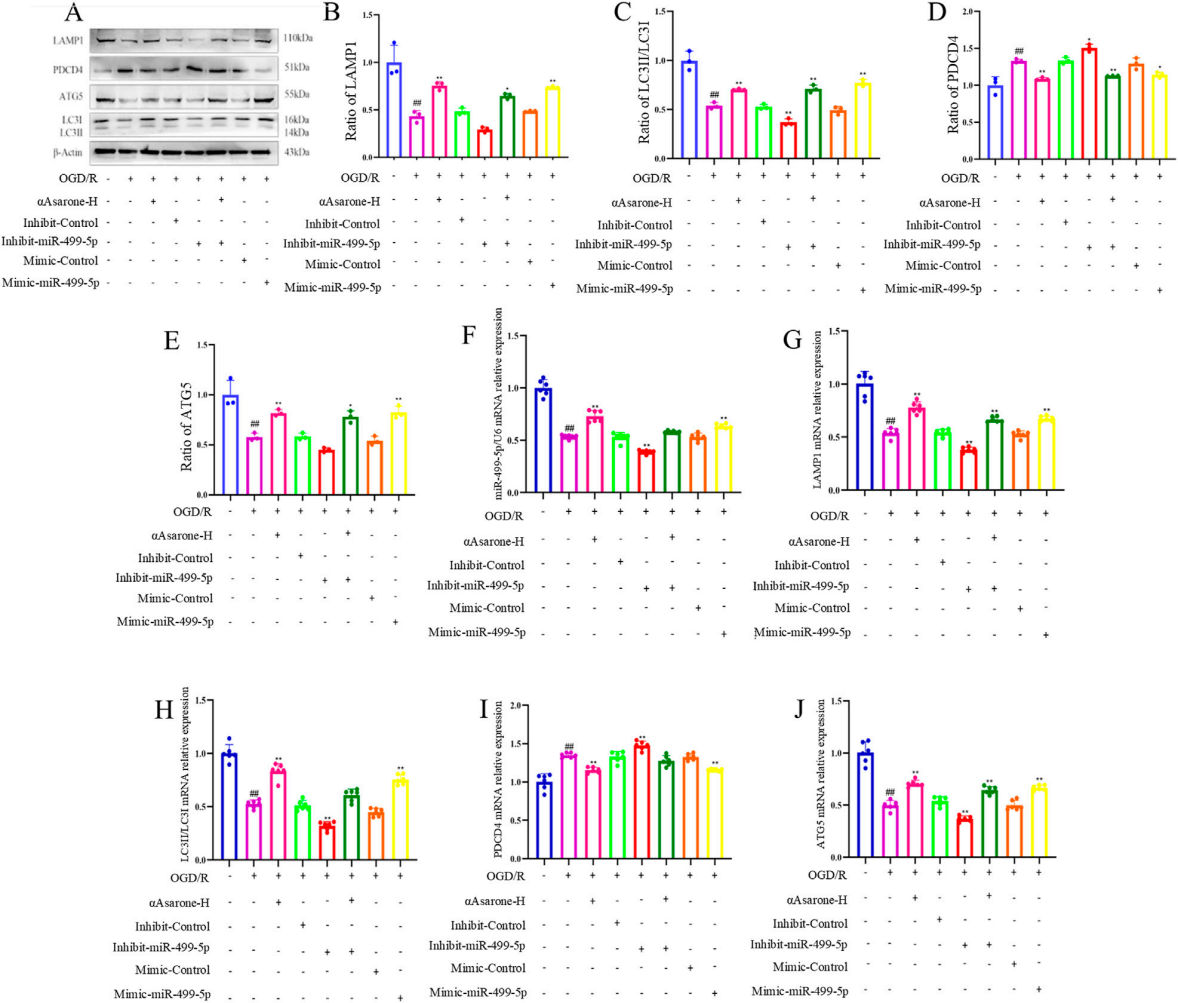
miR-499-5p/PDCD4/ATG5 mediated the activation of autophagy by αAsarone. **(A–J)** The western blots and RT-qPCR of PDCD4, ATG5, LAMP1 and LC3, as well as the quantitative analysis. All data are expressed as mean ± SD, Statistical analysis showed ^##^ *P* < 0.01 vs. the control group, *P < 0.05, **P < 0.01 vs. the OGD/R group.

To investigate the role of αAsarone in the activation of autophagy via the miR-499-5p/PDCD4/ATG5 pathway, miR-499-5p mimics and inhibitors were employed. The results consistently demonstrated that treatment with αAsarone enhanced the autophagy flue, the colocalization of LAMP1 and LC3, and the accumulation of autophagosome. Interestingly, increased autophagy by αAsarone was partially inhibited by the inhibit-miR-499-5p. This inhibition resulted in a reduction in autophagy flue, both colocalization and autophagic body accumulation (Figures 4A–E). Furthermore, αAsarone was found to downregulate the protein and mRNA expression of miR-499-5p, ATG5, and the LC3-II/LC3-I ratio, while simultaneously upregulating PDCD4 expression (Figures 5A–J). These findings indicate that αAsarone enhances autophagy in neurons *in vitro* through a mechanism dependent on miR-499-5p.

### 3.5 αAsarone reduced the neuronal damage in MCAO mice

**FIGURE 6:**
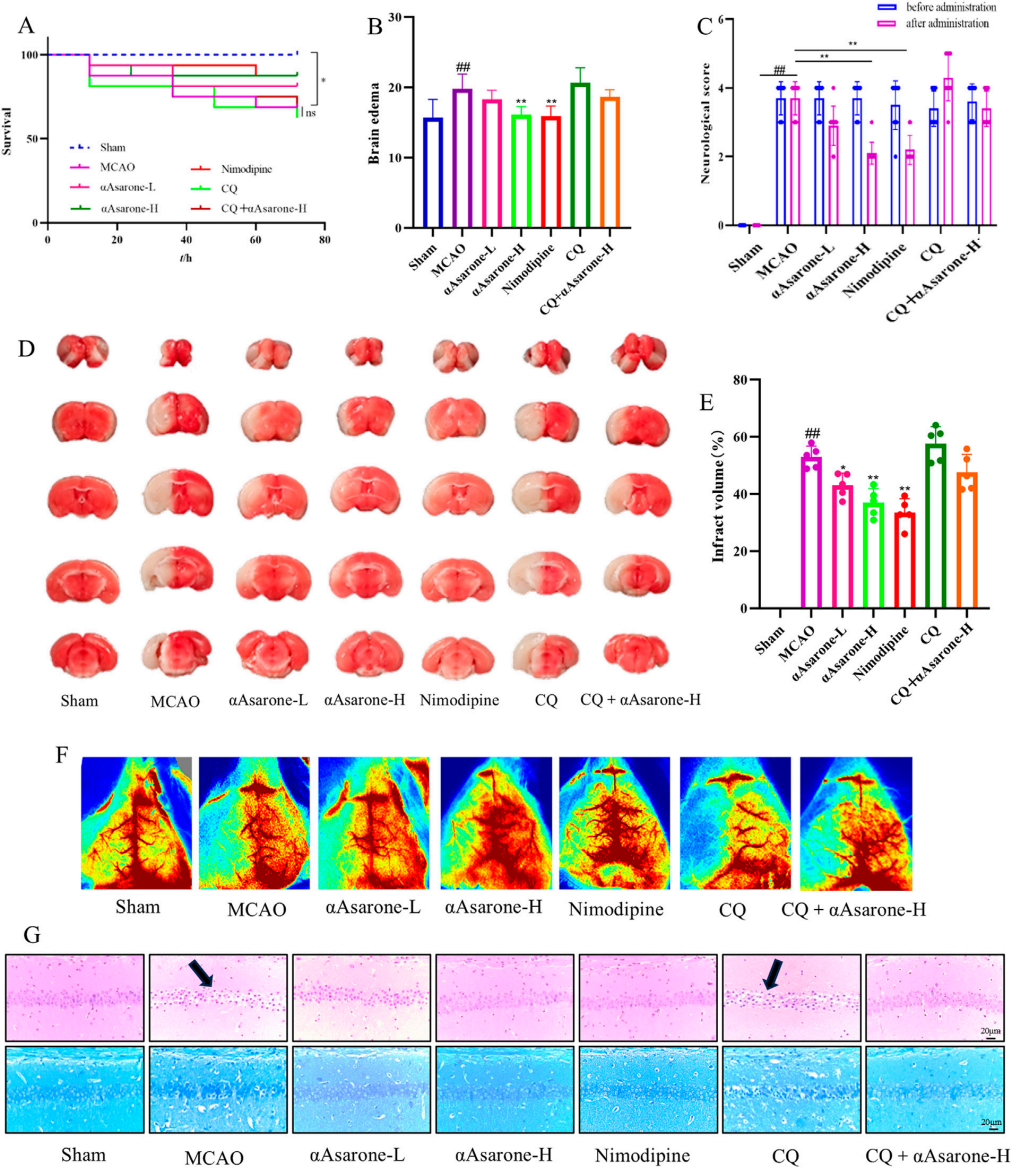
αAsarone reduced the neuronal damage after MCAO. **(A)** Survival curve (log-rank test) (n = 16) **(B)** Brain edema was assessed at 24 h after stroke (n = 10). **(C)** Effect of αAsarone on neurological function score in stroke mice (n = 10). **(D)** TTC staining and cerebral infarct area percentage (n = 5). **(E)** Quantitative analysis of cerebral infarction volume (One-way ANOVA). **(F)** Example LSCI 3 days after MCAO (n = 3). **(G)** Representative photograph of HE staining and Nissl staining in the CAl region of the hippocampus. All data are expressed as mean ± SD, Statistical analysis showed ^##^ *P* < 0.01 vs. the control group, *P < 0.05, **P < 0.01 vs. the OGD/R group.

Here we investigated the protective effects of αAsarone against MACO-induced neuronal injury and examined the mechanisms through which autophagy was caused by αAsarone. The survival rate of mice subjected to MCAO modeling ranged from 60% to 70%, with no significant differences observed in the survival curves among the various treatment groups while there was a significant difference between the normal group and MCAO group (*P* < 0.05) (Figure 6A). In comparison to the model group, both neurological scores and brain edema were reduced in the treatment groups (Figures 6B, C). Infarct size was assessed using vital staining with TTC, and representative images of TTC-stained ischemic brain infarctions are presented (Figure 6D). Normal brain tissue exhibited a deep red color, while infarcted tissue remained unstained. Each treatment group demonstrated a significant reduction in infarct volume compared to the model group (Figure 6E). Laser speckle contrast imaging (LSCI) corroborated the findings from the TTC staining, blood flow increased after αAsarone (Figure 6F). Additionally, examination of H&E and Nissl staining revealed a reduction in neuron numbers within the model group, which was marked by darkened and wrinkled staining of the cells. In comparison, the αAsarone groups exhibited notable enhancements, including a rise in neuron count, clearer cytoplasmic details, and more robust cellular morphology (Figure 6G). Furthermore, as an autophagy inhibitor, CQ was found to block the protective effect of αAsarone.

### 3.6 αAsarone ameliorated neuronal autophagy through miR-499-5p/PDCD4/ATG5

**FIGURE 7:**
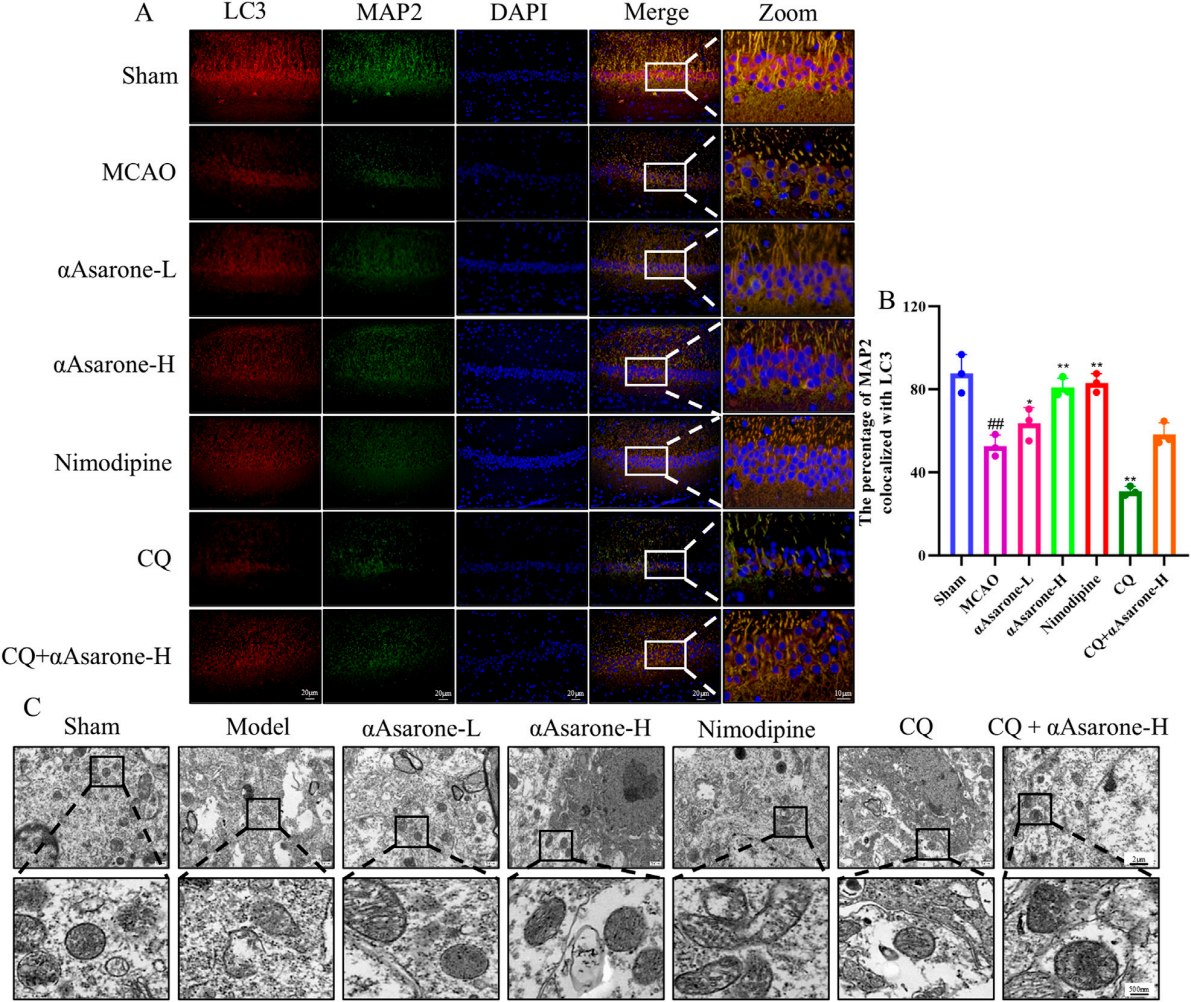
αAsarone ameliorated neuronal injury through miR-499-5p/PDCD4/ATG5 mediated neuronal autophagy. **(A)** Representative immunofluorescence staining for DAPI, LAMP1 and LC3 in the hippocampal neurons. **(B)** The percentage of MAP2 colocalized with LC3. **(C)** The representative electron micrographs in the CA1 region of hippocampus were observed by TEM. The black box represents the autophagosome. All data are expressed as mean ± SD, Statistical analysis showed ^##^ *P* < 0.01 vs. the control group, *P < 0.05, **P < 0.01 vs. the OGD/R group.

**FIGURE 8:**
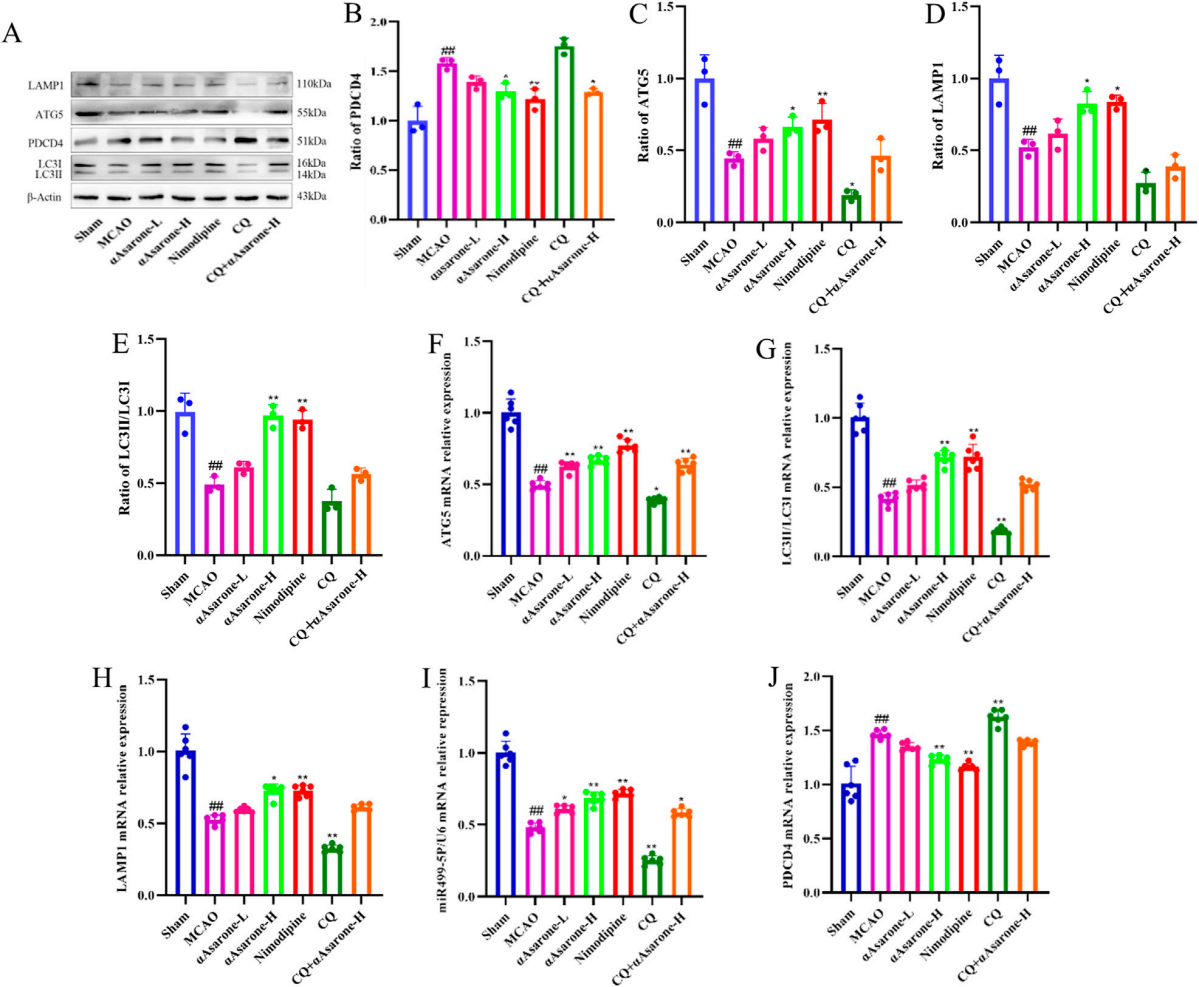
αAsarone ameliorated neuronal injury through miR-499-5p/PDCD4/ATG5 mediated neuronal autophagy. **(A–J)** The western blots and RT-qPCR of PDCD4, ATG5, LAMP1 and LC3, as well as the quantitative analysis. All data are expressed as mean ± SD, Statistical analysis showed ^##^ *P* < 0.01 vs. the control group, *P < 0.05, **P < 0.01 vs. the OGD/R group.

To investigate the impact of αAsarone on the neuronal autophagy through miR-499-5p/PDCD4/ATG5 pathway, we performed immunofluorescence staining and electron microscopy in mice after MCAO. Treatment with αAsarone significantly increased the colocalization of MAP2 and LC3, indicating enhanced autophagy. In contrast, Pretreated with CQ reduced this colocalization, suggesting that CQ inhibited the effects of αAsarone (Figures 7A, B). Furthermore, post-MCAO administration of αAsarone resulted in a significant increase in the number of autophagosomes, which was partially reversed by CQ pretreatment (Figure 7C). Western blot and RT-qPCR analyses demonstrated that αAsarone decreased PDCD4 levels while increasing the ratios of LC3-II/LC3-I, LAMP1, miR-499-5p, PDCD4, and ATG5. Conversely, CQ administration reduced the expression of autophagy-related proteins. However, the combination of CQ and αAsarone resulted in an increased expression level of these proteins (Figures 8A–J). Overall, our findings suggest that αAsarone promotes autophagy, thereby providing neuroprotection through the miR-499-5p/PDCD4/ATG5 pathway.

## 4 Discussion

Long-term disability and death resulting from stroke impose a significant burden on global healthcare systems, results from disrupt blood flow to the brain and cause irreversible neurological damage (Saini et al., 2021; Liu H. et al., 2023). Timely intravenous thrombolysis and intravascular thrombectomy represent the primary interventions for early ischemic stroke; however, their clinical application is constrained by a limited treatment window and stringent indications. Certain traditional oriental herbal medicines, including *acorus calamus*, have been shown to enhance cognitive function. A key active compound found in the rhizomes of these plants is αAsarone (Lee et al., 2018; Zhang et al., 2021). Previous research has shown αAsarone, possesses potential protective effects against stroke (Zhang et al., 2021). In this study, αAsarone demonstrated therapeutic efficacy in the MCAO/R and OGD/R models, as evidenced by significant reductions in neuronal apoptosis, as well as improvements in cell proliferation and tissue morphology. Notably, the therapeutic effects were most pronounced at concentrations of 10, 40 mg/kg *in vivo* and 5, 10, and 20 μM *in vitro*.

The autophagy pathway, a highly conserved self-eating catabolic pathway for the degradation of misfolded proteins or damaged organelles (Hwang et al., 2021). During cerebral ischemia, reduced blood flow, along with a subsequent lack of oxygen, glucose, and other nutrients, resulting in protein misfolding and formation of aggregates, and accumulation of the cell membrane and lipid particles. While, autophagy could eliminate these damaged components, widely accepted to reduce neuronal injury when moderately activated during ischemia (Ahsan et al., 2021; Liu et al., 2024)*. In vitro* OGD/R neuronal models and *in vivo* MCAO animal models have been extensively utilized to simulate ischemic injury, with evidence indicating that the autophagy pathway is participated in these models (Xu et al., 2021; Liu M. et al., 2023). For example, Li et al. (2021) reported that the promotion of mitochondrial autophagy in ischemic neurons and the inhibition of their apoptosis is facilitated by stilbene glycoside. Moreover, Liu et al. (2024) indicate that berberine’s neuroprotective effects against ischemic stroke are achieved through the enhancement of autophagic flux within neurons. Thus, autophagy may have a significant role in reducing neuronal damage in the OGD/R model. As anticipated, the activation of autophagy by αAsarone was evidenced by percentage of colocalization of LAMP1 and LC3 in the cytoplasm observed by fluorescence microscopy, autophagosomes under electron microscopy, and enhanced autophagic flux. It is noteworthy that the ratio of LC3-II/I and LAMP1 increased significantly in the group treated with αAsarone. Besides, CQ and αAsarone combination treatment inhibited the expression of LC3-II/I and LAMP1 *in vivo*. These data indicate that αAsarone alleviates neuronal damage by promoting autophagy. Interestingly, RT-qPCR detection showed that the expression of miR-499-5p increased after αAsarone treatment, proposing that the neuroprotective effects of αAsarone on OGD/R cells might be linked to the activation of autophagy through the mediation of miR-499-5p. This discovery motivated us to explore the possibility that αAsarone could mitigate stroke by stimulating autophagy that is mediated by miR-499-5p.

Numerous studies have demonstrated that miR-499-5p serves multifunctional roles in various brain diseases, including alleviates neurocyte apoptosis and reactive oxygen species production (Martins et al., 2022; Zhou et al., 2022).Our study reports a change in miR-499-5p expression within HT22 cells grown under OGD/R conditions and in mice that underwent stroke induced by MCAO. Specifically, miR-499-5p was found to be downregulated in both the OGD/R HT22 cells and the brain tissues of MCAO-exposed mice. It is possible that αAsarone have shown protective effect can be compared with mimic-miR-499-5p *in vitro*. These findings strongly indicate that αAarone may enhance neuronal autophagy and mitigate neuronal apoptosis during stroke injury by upregulating miR-499-5p levels. To further elucidate the molecular mechanism of αAsarone in stroke treatment, we predicted the targeted downstream factor of miR-499-5p using Targscan. PDCD4, which is expressed in neurons and regulated by miRNAs, has been predicted to be targeted by miR-499-5p and cause its downregulation. A multitude of studies has shown that the modulation of PDCD4 can mitigate cellular damage and improve the survival rates of neuronal cells following a stroke (Lu et al., 2020; Zheng et al., 2020; Ren et al., 2021). The results of our Dual-Luciferase reporter assay showed that miR-499-5p could target PDCD4 and negatively regulated its expression. ATG5 is an important autophagy-related protein that plays a crucial role in the initiation of autophagosome formation, the regulation of autophagy by PDCD4 is influenced by ATG5 (Zheng et al., 2019). Interestingly, transfected with inhibit-miR-499-5p damaged the inhibitory effect of αAsarone on PDCD4 expression and promoting effect on ATG5 expression, thereby mitigating the protective effects of αAsarone against neuronal injury. These findings suggest that αAsarone alleviates neuronal injury by facilitating autophagy via miR-499-5p/PDCD4/ATG5 signaling pathway in stroke.

Although αAsarone demonstrated potential to be a novel and effective drug to treat stroke, the present study had several limitations. First, our study is limited by the deficiency of data on changes in miR-499-5p, PDCD4, and ATG5 expression before and after αAsarone treatment in clinical samples. Future research will focus on examining clinical samples to further validate the molecular indicators associated with this mechanism. Second, to elucidate whether autophagy inhibition has a direct effect, CQ, a late-stage autophagy inhibitor that blocks lysosomal function and consequently impacts the fusion stage, has been applied. However, it is essential to assess autophagy objectively at all stages. For instance, 3-methyladenine is necessary to inhibit class II phosphoinositide 3-kinases, which primarily affects the nucleation stage of autophagy. Finally, we aim to prolong the intervention cycle for a mouse stroke model, focusing more on the effects of αAsarone at various time intervals, and will investigate the dynamic alterations of several observation indicators in upcoming studies.

## 5 Conclusion

Taken together, the present results demonstrate that αAsarone exerts neuroprotection via regulating neuronal autophagy following stroke. The current study provides novel insights that targeting the novel miR-499-5p/PDCD4/ATG5 pathway could be a beneficial strategy. In summary, αAsarone treatment might be a rational strategy for stroke.

## Data Availability

The original contributions presented in the study are included in the article/supplementary material, further inquiries can be directed to the corresponding authors.

## Abbreviations

MCAO, middle cerebral artery occlusion; OGD/R, oxygen glucose deprivation/re-oxygenation; RT-qPCR, Real-time reverse transcription-quantitative PCR; PBS, phosphate buffered saline; TBST, tris buffered saline tween; TTC, 2,3,5-triphenyltetrazolium chloride; TEM, transmission electron microscope; DAPI, Enhanced chemiluminescence; CQ, chloroquine.
